# IMSE: interaction information attention and molecular structure based drug drug interaction extraction

**DOI:** 10.1186/s12859-022-04876-8

**Published:** 2022-08-14

**Authors:** Biao Duan, Jing Peng, Yi Zhang

**Affiliations:** 1 Wuhan University of Technology, GongDa Road, Wuhan, China; 2 Intelligent Bioinformatics Laboratory, Wuhan University of Technology, GongDa Road, Wuhan, China

**Keywords:** Drug–drug interactions, Side efects, Dug molecular structure

## Abstract

**Background:**

Extraction of drug drug interactions from biomedical literature and other textual data is an important component to monitor drug-safety and this has attracted attention of many researchers in healthcare. Existing works are more pivoted around relation extraction using bidirectional long short-term memory networks (BiLSTM) and BERT model which does not attain the best feature representations.

**Results:**

Our proposed DDI (drug drug interaction) prediction model provides multiple advantages: (1) The newly proposed attention vector is added to better deal with the problem of overlapping relations, (2) The molecular structure information of drugs is integrated into the model to better express the functional group structure of drugs, (3) We also added text features that combined the T-distribution and chi-square distribution to make the model more focused on drug entities and (4) it achieves similar or better prediction performance (F-scores up to 85.16%) compared to state-of-the-art DDI models when tested on benchmark datasets.

**Conclusions:**

Our model that leverages state of the art transformer architecture in conjunction with multiple features can bolster the performances of drug drug interation tasks in the biomedical domain. In particular, we believe our research would be helpful in identification of potential adverse drug reactions.

## Background

Polypharmacy, the concurrent administration of multiple drugs, has been increasing among patients in recent years [1–3]. When administering multiple drugs, interactions might arise among them, often termed drug–drug interactions (DDI). The intended effect of a drug may therefore be altered by the action of another drug. These effects could lead to drug synergy [4], reduced efficacy or even to toxicity [5]. Thus, DDI interaction extraction is an important step towards improved patient treatment and safety.

Traditionally, doctors have obtained the latest information on DDI from two main sources: reading numerous biomedical papers to learn about DDI or querying DDI from biomedical databases. In the biomedical field, the number of biomedical literature has been increased rapidly. Obviously, reading a large number of papers is inefficient. As for biomedical databases, it seems possible, but in the consideration of the quantity of the biomedical literature, it requires a lot of resources to update and revise a professional database manually. So, two of these methods are not ideal for obtaining DDI.

The DDI extraction task [6] aims to extract DDI from free texts in the biomedical field. DDIExtraction 2013 task seeks to classify each DDI candidate according to one of five types (“Advise”, “Effect”, “Mechanism”, “Int” and “False”).

In the early days, people often adopt the pattern-based methods and feature-based machine learning methods [7, 8], but methods based on pattern requires the annotator to have certain domain knowledge, and the main drawback of this method is both time-consuming and inefficient.

Deep learning is the most widely applied and effective method to solve this problem at present, mainly including CNN-based methods [9–15], RNN-based methods [16–22] and currently the best methods based on pre-training. In general, RNN is suitable for NLP applications due to cyclic connections [23, 24], but RNN has the problem of explosion and vanishing gradient [25]. To address these problems, the long term short term memory (LSTM) [26, 27] unit and the gated recurring unit (GRU) [28] network were proposed.

In recent years, methods based on pre-training [29, 30] have achieved good results. Lee et al. introduced BioBERT (bidirectional encoder representations from transformers for biomedical text mining) to improve DDI extraction [31], the authors pre-trained BioBERT on PubMed abstracts (PubMed) and PubMed Central full-text articles (PMC). Boukkouri et al. put forward a new variant of BERT [32], it is completely abandoned the chunk system, and use a character-CNN module instead of by query their characters to represent whole words. Recently, Sun et al. [33] further improved the extraction effect of DDIExtraction 2013 task by introducing Gaussian vector and other external knowledge on the basis of BioBERT.

**Fig. 1:**
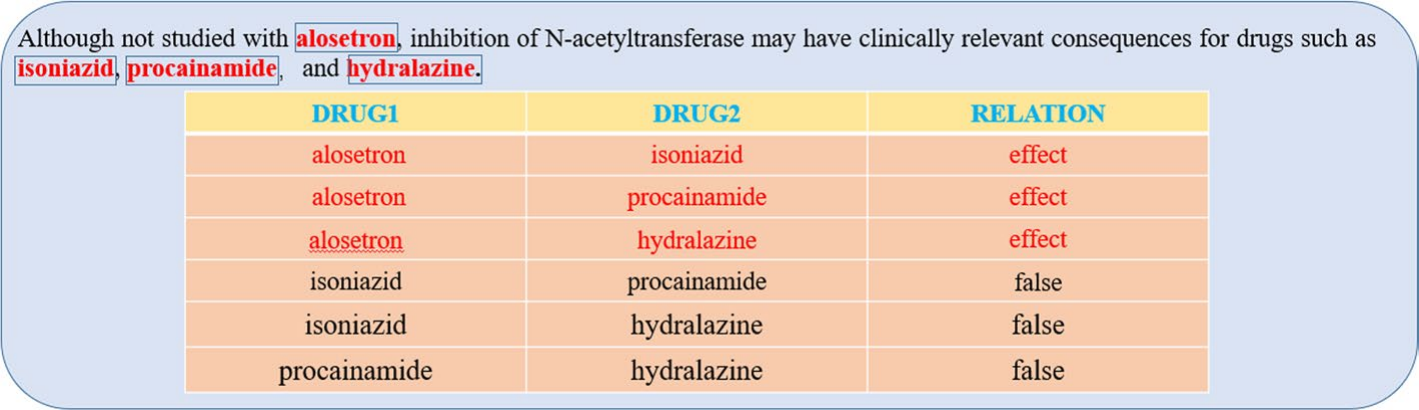
An example of overlapping relationships. Drugs entities are labeled. This example shows that there are multiple drug entities and multiple interactions in a sentence

The above mentioned solutions come with some drawbacks. First, as shown in Fig. 1, in the sentence that contains the DDIs, there are multiple complex drug drug interactions. For example, drug ‘alosetron’ and the other three drugs (‘isoniazid’, ‘procainamide’, ‘hydralazine’) all have effects. We called this relationship overlap, for this kind of complex relationship overlap, the above methods do not have good solutions. The newly proposed attention vector is designed to better deal with the problem of overlapping relationships. Second, there is a plethora of drug feature information available for many approved drugs, including molecular structure, drug SMILES, and more. All of the above methods ignore this additional but very useful information. Therefore, we obtained the SMILES molecular formulas of the drugs through the DrugBank [34] database, and transformed the SMILES molecular formulas into the fingerprints and adjacency matrices through the RDKit toolkit [35] to get drug molecular features. In addition, the location of drug entities in the text is also very helpful to extract specific drug relationships. For this purpose, we introduce T-distribution and Chi square distribution to obtain the sentence feature which focus on drug entities.

Rest of our work is organized as follows. We first discuss our approach in detail i.e system architecture, then experimental setup, training and evaluation metrics. This is followed by a discussion of the experimental results and in the end we make a conclusion.

## Methods

**Fig. 2:**
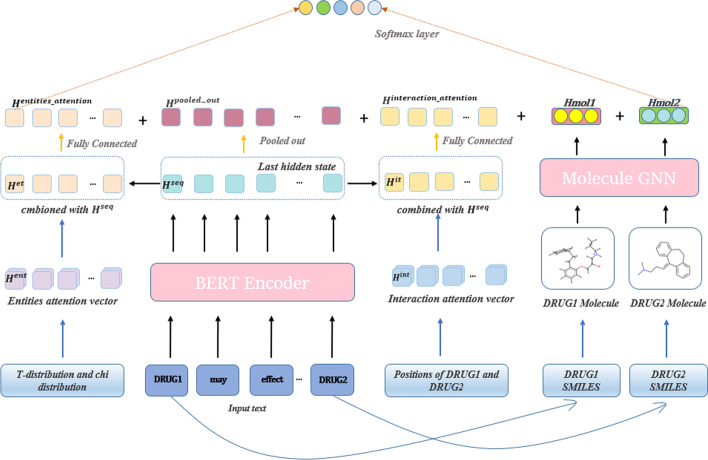
Architecture of the proposed model. Our model is mainly distributed in four parts. First, BioBERT is used to encode the input sentences, and meanwhile, molecular graph neural network is used to encode the drug structure. Then, Interaction attention vector and Entities attention vector are respectively generated to combine the output of Biobert. Finally, all the obtained information is sent to the classifier for prediction

In this section, we introduce our system architecture (Fig. 2) and explain different modules it invokes in a sequential manner. Figure 2 shows the architecture of our approach. Our model is divided into four parts. First we use Biobert to encode the input sentence, and get the last hidden state (${H}^{seq}$) of Biobert. Then, we generate attention vector according to the positions of DRUG1 and DRUG2 and obtained ‘interaction features’ which are helpful to identify overlapping relation, and we got ‘entities attention features’ which focus on entities by introducing Chi-square distribution and T-distribution. Last, we obtained the drug structure according to the DrugBank database and the RDKit tool library, and we use molecular graph neural network [36] to generate high quality molecular representations. Finally, we combined all the information to make the classification of DDI. We will introduce our model and method in detail in the following content.

### Data pre-processing

#### Drug mask

For the two drug entities in the sentence that need to classify the type of relationship, ‘DRUG1’ and ‘DRUG2’ were used to replace them, and for other drug entities in the sentence, ‘DRUGOTHER’ was used to replace them.

#### Over-sampling and under-sampling

DDI 2013 dataset has long-tail distribution phenomenon. Therefore, under-sampling and over-sampling methods are adopted to optimize the training set. Compared with the original data, a large number of repeated negative samples are filtered out after undersampling while oversampling can significantly increase the number of sparse samples.

**Fig. 3:**
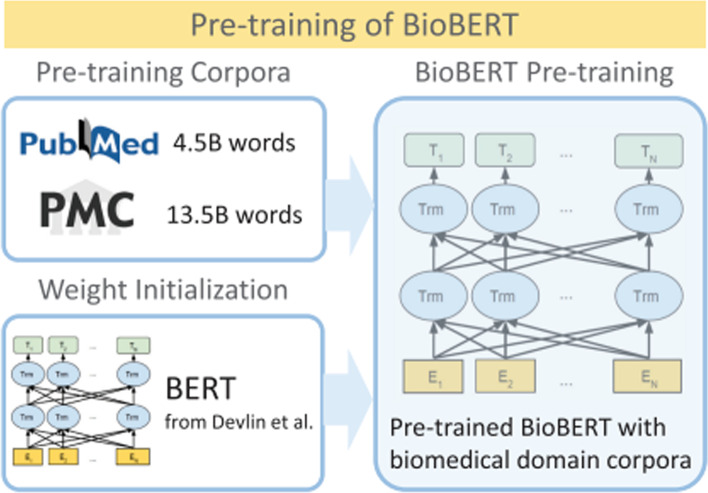
Overview of the pre-training of BioBERT [31]. BioBERT is a domain-specific language representation model pre-trained on large-scale biomedical corpora. With almost the same architecture across tasks, BioBERT largely outperforms BERT and previous state-of-the-art models in a variety of biomedical text mining tasks when pre-trained on biomedical corpora

### Sentence encoder

The goal of this component is to obtain the context-aware representation of each token in a sentence. Given the impressive performance of recent deep transformers trained on variants of language modeling, we utilize the BioBERT model as the sentence encoder. The BioBERT was pre-trained on a lot of PubMed abstracts and PubMed Central full-text articles, and see Fig. 3 for the overview of the pre-training. Given an sequence ($S=\{x_{1},x_{2},\dots ,x_{n}\}$) as input, BioBERT can be formulated as follows:

1 $$\begin{aligned}&h_{i}^{0}={\varvec{W}}_{\varvec{e}} x_{i}+{\varvec{W}}_{\varvec{b}} \end{aligned}$$

2 $$\begin{aligned}&{h}_{i}^{l}=transformer\_block\left( h_{i}^{l-1}\right) \end{aligned}$$

3 $$\begin{aligned}&t_{i}^{L}=h_{i}^{L} \end{aligned}$$

4 $$\begin{aligned}&{H}^{pooled\_out}=h_{C L S}^{L} \end{aligned}$$

where *xi* is the *i*-th token, *L* is the total number of layers for BERT, *l* (1 $<l<L$) is the *l*-th layer. Equation (1) indicates input embeddings, Eq. (3) denotes the representation of the *i*-th token, and Eq. (4) denotes the representations of the sequence. The transformer_block in Eq. (2) contains multi-head attention layers, fully connected layers, and the output layer. Furthermore, the parameters ${\varvec{W}}^{e}$, ${\varvec{W}}^{b}$, and $transformer\_block$ are pre-trained on large-scale corpora using two unsupervised pre-training tasks, masked language model and next sentence prediction. The output of the BioBERT model is the context-aware embedding of tokens, and is denoted as ${\varvec{H}}^{seq} \in R^{n \times d}$, where *n* is the sentence length (including [CLS] and [SEP], two special start and end markers), and *d* is the number of hidden units in the BERT model.

### Interaction attention vector

In this part, we will introduce the interaction attention vector. As shown in the sentence in Fig. 1, if the two drugs have some interaction, they will be far apart in the sentence, whereas if they do not interact, they will be closer together, suggesting that the important information characterizing the interaction is often between the two entities. In addition, we found this pattern in the vast majority of biological texts. So when dealing with overlapping relationships we should focus on the information between the current pair of drugs. In Table 1, we give the statistics of the interaction information between entities in SEMEVAL-2013 DATA SET. We have performed the statistics for the training set, validation set and test set separately, and the results show that the large batches of data among the datasets fit this pattern. That is to say we should pay more attention to the content between entities. In the following we will explain in detail how to generate the interacting attention vector and how to use it.

**Table 1:** Data of interaction information between entities as a percentage of total data

Relation	Train	Dev	Test	All
Mechanism	0.89	0.90	0.92	0.90
Effect	0.87	0.91	0.88	0.89
Advice	0.85	0.83	0.85	0.84
Int	0.84	0.86	0.86	0.85

We define a high weight range and a lower weight range:

5 $$\begin{aligned}&{\varvec{high}}\, {\varvec{weight}}\, {\varvec{range}}=\left( {\varvec{h}}_{w 0}=0.9, {\varvec{h}}_{w 1}=1.1\right) \end{aligned}$$

6 $$\begin{aligned}&{\varvec{low}}\, {\varvec{weight}}\, {\varvec{range}}=\left( {\varvec{l}}_{w 0}=0.3, {\varvec{l}}_{w 1}=0.5\right) \end{aligned}$$

We assign the high weight to the information between the two drugs, and lower weight to the rest of the sentence. In order to keep this range of weights elastic, we add an oscillation factor $\sigma$ to the weight range. The weight after adding the shock factor are as follows, ${\varvec{W}}_{hign }$ is the weight range we will assign to the information between the two drugs, ${\varvec{W}}_{low }$ is the weight range we will assign to the rest of the sentence:

7 $$\begin{aligned}&{\varvec{W}}_{high }=\left[ {\varvec{h}}_{w 0}-\sigma , {\varvec{h}}_{w 1}+\sigma \right] \end{aligned}$$

8 $$\begin{aligned} {\varvec{W}}_{low }=\left[ {\varvec{l}}_{w 0}-\sigma , {\varvec{l}}_{w 1}+\sigma \right] \end{aligned}$$

where $\sigma$ is the oscillation factor (here we take 0.1).

We define a sentence $S=\{x_{1},x_{2},\dots ,x_{n}\}$, the final Interaction attention vector we proposed is defined as follows:

9 $$\begin{aligned} {\varvec{H}}^{int}={\varvec{W}}_{low }\sum _{start}^{j-1}x_{i}+{\varvec{W}}_{high }\sum _{j}^{k} x_{i}+{\varvec{W}}_{low } \sum _{k+1}^{end} x_{i} \end{aligned}$$

where *start* and *end* means the start and end of a sentence, *j* and *k* means the positions of head enity and tail enity in the sentence, ${\varvec{H}}^{int}$ is the final Interaction attention vector, and its visual representation is as follows:

**Fig. 4:**
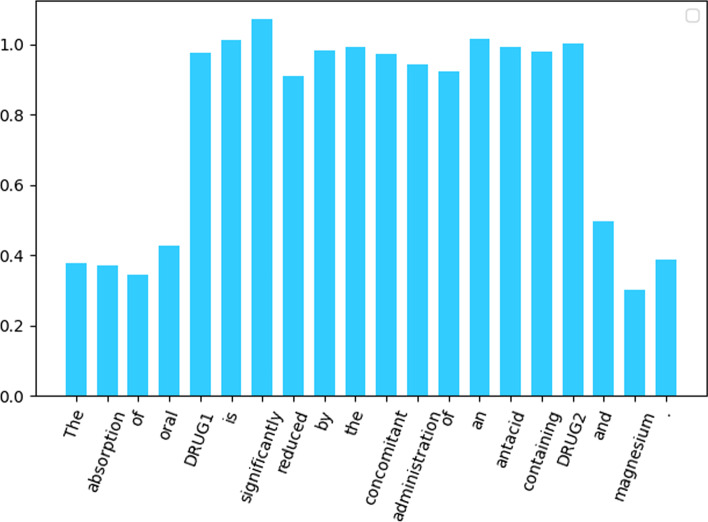
Visual representation of the Interaction attention vector. The horizontal axis represents each token in the sentence, and the vertical axis represents the different weights assigned to them according to the Interaction attention vector. The information between the two drug entities is important that it is given a high weight, and the rest is given a low weight

The output of the BERT model ($H^{s e q} \in R^{d \times 1}$) is the context-aware embedding of tokens. Then we do a matrix operation with ${\varvec{H}}^{int}$, the output of this step is like Fig. 4, each word embedding of ${\varvec{H}}^{seq}$ is given a different weight, the formulas are as follows:

10 $$\begin{aligned} {\varvec{H}}^{seq}= {\varvec{Transformer}}\_{\varvec{block}}\left( {input\_sentence}\right) \end{aligned}$$

11 $$\begin{aligned} {\varvec{H}}^{i t}={\varvec{H}}^{seq} \otimes {\varvec{H}}^{int} \end{aligned}$$

where ${input\_sentence}$ is the original input of the sentence, ${\varvec{H}}^{i t}$ (${\varvec{H}}^{i t} \in R^{d \times 1}$) is the synthesis vector obtained after the fusion of ${\varvec{H}}^{int}$ and ${\varvec{H}}^{seq}$, $\otimes$ means matrix multiplication.

We also apply the average operation on the comprehensive vector representation ${\varvec{H}}_{i t}$:

12 $$\begin{aligned} {\varvec{H}}^{interaction\_attention}={\mathbf{W}}_\text {int}\left[ {\tanh } \left( \frac{1}{k-j+1} \sum _{j}^{k} {\varvec{H}}^{i t}\right) \right] +{\mathbf{b}}_{\mathbf{int}} \end{aligned}$$

where ${\varvec{H}}^{interaction\_attention}$ is the output after the average processing of ${\varvec{H}}^{i t}$ and a fully connected layer, *j* and *k* are the positions of first drug and second drug.

For the hidden state output ${\varvec{H}}^{seq}$, we first get pooled output from it, then add an activation operation and a fully connected layer, which is formally expressed as:

13 $$\begin{aligned} {\varvec{H}}^{pooled\_out}={\mathbf{W}}_{\mathbf{0}}\left( \tanh \left( {pooled}\_{out}({\varvec{H}}^{seq}\right) )\right) +{\mathbf{b}}_{\mathbf{0}} \end{aligned}$$

where matrices ${\mathbf{W}}_{\text{int} } \in R^{n \times 1}$, ${\mathbf{W}}_{\text{0 }} \in R^{n \times 1}$ are weight matrices and they have the same dimensions, ${\mathbf{b}}_{\mathbf{int}}$, ${\mathbf{b}}_{\mathbf{0}}$ are bias of neural network.

### Entities attention features

In this study, we introduce the Chi-square probability distribution and T-distribution to enhance the weights of the target entity and its adjacent words, so that the model can learn the local structure of entities. We refer to these two modified distributions together here as the Entities attention vector. The Chi-square probability density function is:

14 $$\begin{aligned} f(x ; k)=\left\{ \begin{array}{ll} \frac{x^{(k / 2-1)} e^{-x / 2}}{2^{k / 2} \Gamma \left( \frac{k}{2}\right) }, &\quad x>0 \\ 0, &\quad \text{other } \end{array}\right. \end{aligned}$$

the Chi-square probability distribution function is:

15 $$\begin{aligned} P(x)_{c}=\int _{-\infty }^{x} f(x ; k) d x-\int _{-\infty }^{x-t} f(x ; k) d x \end{aligned}$$

the T probability density function is:

16 $$\begin{aligned} f(x ; n)=\frac{\Gamma \left( \frac{n+1}{2}\right) }{\sqrt{n \pi } \Gamma \left( \frac{n}{2}\right) }\left( 1+\frac{x^{2}}{n}\right) ^{\frac{n+1}{2}} \end{aligned}$$

the T probability distribution function is:

17 $$\begin{aligned} P(x)_{t}=\int _{-\infty }^{x} f(x ; n) d x-\int _{-\infty }^{x-w} f(x ; n) d x \end{aligned}$$

where *x* is a real value, $\Gamma$ is the Gamma function , *k* is the degree of freedom in Chi-square distribution, *n* is the degree of freedom in T distribution and *t* is the step size of the Chi-square function, *w* is the step size of the T function.

We first get the values of the distribution and then locate the position of the two entities in the sentence. We map 15% before the first entity according to the rule of right-to-left position and high-to-low value, and 25% after the first entity according to the rule of left-to-right position and high-to-low value, and then do a symmetric operation on the right entity.

**Fig. 5:**
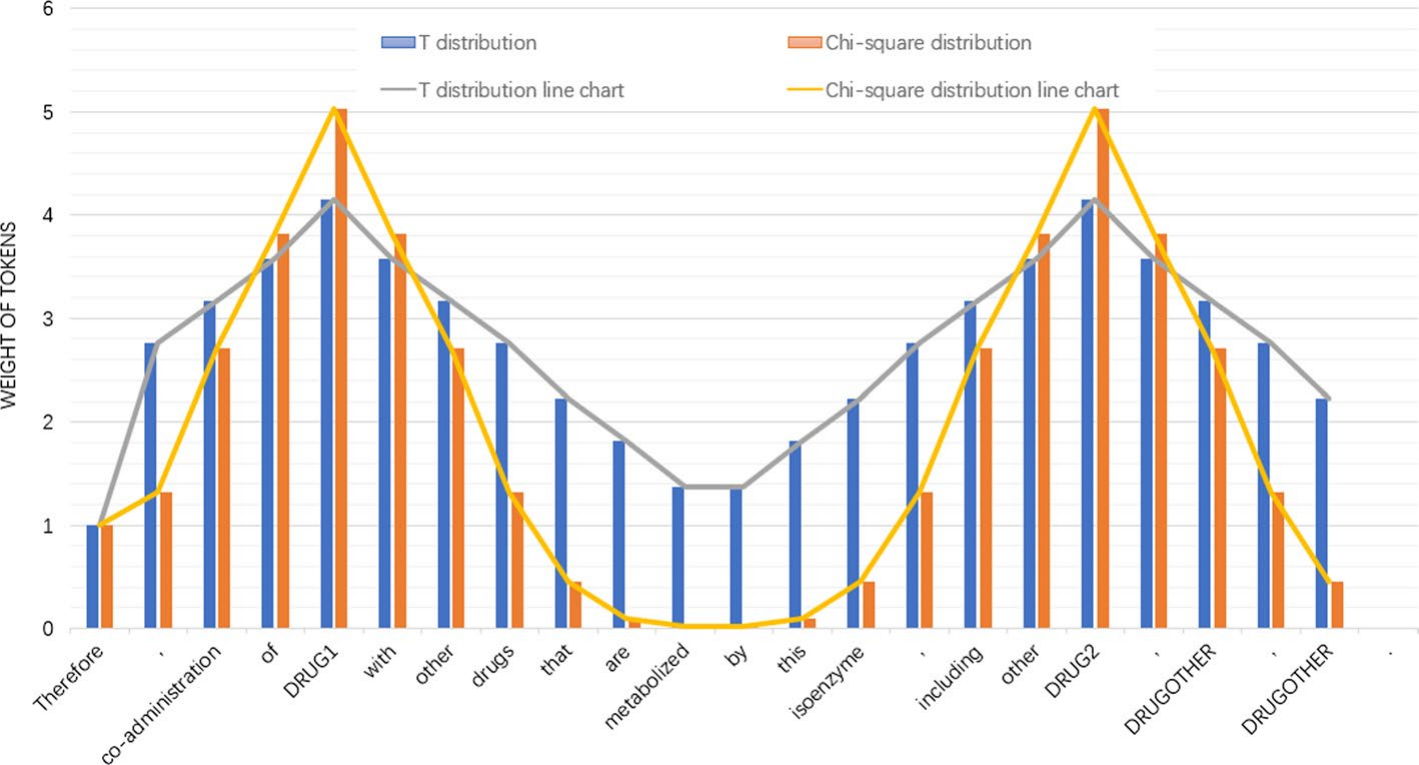
Visual representation of the Entities attention vector. The horizontal axis represents each token in the sentence, and the vertical axis represents the different weights assigned to them according to the Entities attention vector (combination of modified T-distribution and Chi-square distribution)

Finally, we obtained the Entities attention vector ${\varvec{H}}^{ent}$ as Fig. 5, then we do a matrix multiplication with the last hidden state output of BioBERT (${\varvec{H}}^{seq}$), and add an activation operation and a fully connected layer. The formula is as follows:

18 $$\begin{aligned}&{\varvec{H}}^{e t}={\varvec{H}}^{ent}\otimes {\varvec{H}}^{seq} \end{aligned}$$

19 $$\begin{aligned}&{\varvec{H}}^{entities\_attention}={\mathbf{W}}_{\mathbf{ent}}\left( {\tanh } \left( {\varvec{H}}^{e t}\right) \right) +{\mathbf{b}}_{\mathbf{ent}} \end{aligned}$$

where ${H}^{ent}$ is the Entities attention vector, ${\varvec{H}}^{e t}$ ($H^{e t} \in R^{1 \times d}$) is the synthesis vector obtained after the fusion of ${\varvec{H}}^{ent}$ and ${\varvec{H}}^{seq}$, and ${\varvec{H}}^{entities\_attention}$ is the output after an activation operation and a fully connected layer, ${\mathbf{W}}_{\text {ent }} \in R^{n \times 1}$ is the weight matrix, ${\mathbf{b}}_{\mathbf{ent}}$ is the bias of fully connected layer.

### Molecular structure

**Fig. 6:**
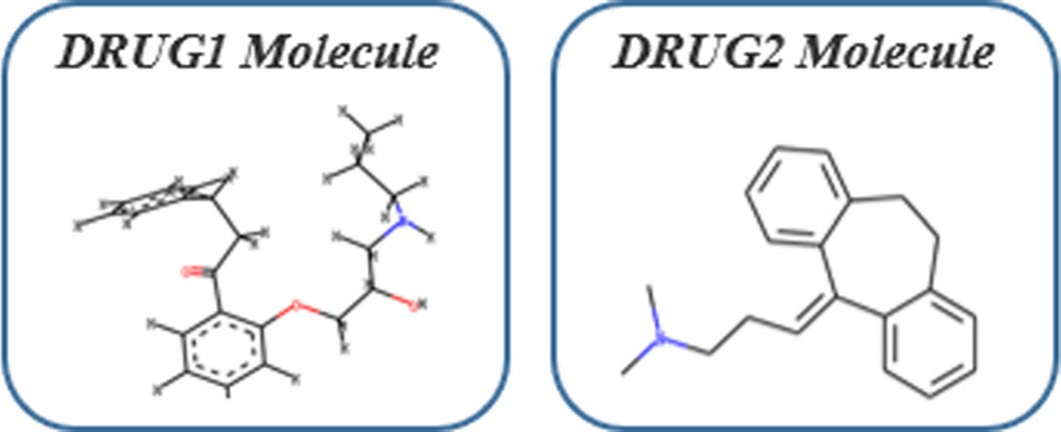
Examples of drug molecular structure. First we can obtain the corresponding drug molecular formula through DrugBank database, and then convert it into a molecular map using RDkit, a tool in chemistry

DrugBank is a freely available drug database containing more than 10,000 drugs. According to the name of drugs, we first found the SMILES formulas of the corresponding drugs in the DrugBank database, and then extracted the corresponding drug structures using the extraction method provided by Tsubaki et al [36]. Figure 6 shows the molecular structure of the drug that we obtained. We use *r*-radius subgraphs which are induced by the neighboring vertices and edges within radius r from a vertex. This *r* is the number of hops from the current vertex to the nearby vertexes, and we take radius *r* 1 here.

We use molecule graph neural network to encode molecular graph structures. GNNs convert a drug molecule graph *G* into a fixed size vector. We represent atoms as nodes and bonds as edges in the graph. Then we feed the fingerprint vectors into the molecule graph neural network which takes the input ngerprint vectors as the initial vectors and updates them according to the structure of the molecular graph. We dene the vector of the *i*-th atom in a drug molecule as ${\varvec{m}}_{i}$ and the set of its neighboring atoms as $N_{i}$. The vector ${\varvec{m}}_{i}$ is updated in the $\ell$-th step as follows:

20 $$\begin{aligned} {\varvec{m}}_{i}^{\ell }={\varvec{m}}_{i}^{\ell -1}+\sum _{j}^{j \in N_{i}} f\left( {\varvec{\omega }}_{hidden}^{\ell -1} {\varvec{m}}_{j}^{\ell -1}+{\varvec{b}}_{hidden}^{l-1}\right) \end{aligned}$$

where *f*() denotes a ReLU function. The drug molecular vector is obtained by summing up all the atom vectors and then the resulting vectors are fed into a linear layer.

21 $$\begin{aligned} {\varvec{H}}^{m o l}=f\left( {\varvec{\omega }}_{output} \sum _{i}^{M} {\varvec{m}}_{i}^{L}+{\varvec{b}}_{output}\right) \end{aligned}$$

where *M* is the number of fingerprints, ${\varvec{H}}^{mol}$ is the output of MGNN, and we thus obtained the molecular structure output of the two drug entities: ${\varvec{H}}^{mol1}$ and ${\varvec{H}}^{mol2}$.

### Softmax layer

In this step, we combine the output obtained earlier to make the fusion representations ${\varvec{H}}$. Then a fully connected neural network is employed to learn the representations ${\varvec{H}}$. Finally, the softmax function is used to calculate the probability *P* belonging to the DDI type *r*:

22 $$\begin{aligned}&{\varvec{H}}= {concat}[{\varvec{H}}^{seq}; {\varvec{H}}^{interaction\_attention}; {\varvec{H}}^{entities\_attention}; {\varvec{H}}^{mol1}; {\varvec{H}}^{mol2}] \end{aligned}$$

23 $$\begin{aligned}&P(r \mid {\varvec{H}})={\text {softmax}}\left( {\mathbf{W}}_{\mathbf{*}} {\varvec{H}}+{\mathbf{b}}_{\mathbf{*}}\right) \end{aligned}$$

where ${\mathbf{W}}_{\mathbf{*}}$ and ${\mathbf{b}}_{\mathbf{*}}$ are weight parameters and bias parameters, and we use the cross-entropy function as the loss function.

### Dataset and experimental settings

DDIExtraction 2013 corpus is a manually annotated drug–drug interaction (DDI) corpus based on the DrugBank database and MEDLINE abstracts. This corpus contains four DDI types for evaluation purposes which are ‘Advice’, ‘Effect’, ‘Mechanism’ , ‘Int’, and a label named ‘False’ which indicates no interaction. We formulate DDI extraction into a multi-class classification problem. We follow DDIEXTRACT-2013 shared tasks (semeval-2013 Task9.2), here we refer to the DDI corpus provided by Asada et al. [15]. Table 2 illustrates the statistics for the instances in the DDIExtraction 2013 dataset.

The task defines the following four interaction labels.

- Mechanism: this type is assigned when a pharmacokinetic mechanism is described in an input sentence.
- Effect: this type is assigned when the effect of the DDI is described.
- Advice: this is assigned when a recommendation or advice regarding the concomitant use of two drugs is described.
- Int (Interaction): this type is assigned when the sentence simply states that an interaction occurs and does not provide any detailed information about the interaction.

**Table 2:** Statistics of SEMEVAL-2013 DATA SET

	Train		Test	
	Drugbank	MEDLINE	Drugbank	MEDLINE
#documents	572	142	158	33
#sentences	5675	1301	973	326
#drug pairs	26,005	1787	5265	451
#positive pairs	3789	232	884	95
#negative pairs	22,216	1555	4381	356
Mechanism	1257	62	278	24
Effect	1535	152	298	62
Advice	818	8	214	7
Int.	179	10	94	2

In the experiments, we employed the PyTorch (https://pytorch.org/) framework to implement our proposed model. For the selection of BERT model, we chose the BioBERT model to encode the input sentences. All of the DDI extraction methods use the standard evaluation measures (precision, recall and F-score) as the evaluation metrics. The F-score is defined as: $F1 = 2PR/(P + R)$.

## Results

**Table 3:** Performance comparison with other state-of-art methods on DDIExtraction 2013 dataset

	Methods	Precision (%)	Recall (%)	F1-score (%)
CNN-based	SCNN [12]	69.1	65.1	67.0
	DCNN [11]	77.2	64.4	70.2
	MCCNN [10]	76.0	65.3	70.2
LSTM-based	DLSTM [18]	72.5	71.5	72.0
	ASDP-LSTM [20]	74.1	71.8	72.9
	Tree-LSTM [37]	77.8	69.6	73.5
	ATT-BLSTM [19]	78.4	76.2	77.3
Pretraining-based	BERT [29]	77.90	77.43	77.66
	BioBERT [31]	81.1	75.3	78.1
	CharacterBERT [32]	79.18	80.38	81.70
	ChemicalBERT + AGGCN [38]	83.96	81.82	82.88
	DESC_MOL [39]	84.69	82.53	83.60
	IMSE (ours)	**85**.**63**	**85**.**17**	**85**.**16**

Bold indicates the highest value of the measured metric in each comparison experiment

### The performances of IMSE on the benchmark datasets and analysis

Table 3 illustrates the experimental results in detail. We compared our model with typical models based on CNN, RNN and pre-training. BioBERT is pre-trained in PubMed Abstracts (PubMed)and PubMed Central Fulle-Text Articles (PMC). CharacterBERT model use a character-CNN module instead of by query their characters to represent whole words. ChemicalBERT + AGGCN (Parallel) is a model combined GCN with ChemicalBERT for DDI task. In general, LSTM-based models achieve better results than CNN-based models because the LSTM structure can handle long text while CNNs focus more on local features. In addition, pre-trained-based models perform better than other methods, for example, the BioBERT model pre-trained with a large amount of biomedical text achieves an F1-score of 78.1%. The experimental results show that our method (IMSE) outperforms all the latest models, with a 7.06% higher F1-score than the baseline model BioBERT, as well as 1.56% and 2.28% higher F1-scores than the latest DESC_MOL and ChemicalBERT + AGGCN, respectively. Compared with other pre-training-based methods, the Interaction attention operation enables the model to focus more on information that facilitates correct classification results, i.e., critical information. The Entities attention operation enables the model to focus more on the drug entity itself and ignore other interfering drugs. Molecular structure as additional information can also provide the model with structural features in addition to text.

### Ablation experiments

In this section, to explore the contribution of each component to overall performance, we performed an ablation study over our proposed model. We did a total of six comparative experiments, and the experimental results are presented in Table 4. As shown in the table, the F1-score of BERT(BioBERT) model when we did not add any operations was 78.1%. Then when we add only Entities attention vector, the F1-score increases by 2.79% compared with BERT, which indicates that adding the Entities attention information is helpful in determining the relationship between the drugs. Next, we evaluated the impact of the Interaction attention vector which we proposed. When the Interaction attention vector is added, the F1-score increases by 5.66% compared with BERT, reached an F1-score of 83.76%. Then we added the Interaction attention vector and Entities attention vector at the same time in the fourth experiment, we label this method as ‘BERT + Int* + Ent*’, as can be seen from the table, the precision score, recall score and F1-score of this method reached 85.54%, 83.56% and 84.47% respectively, it is fully demonstrated that both the information of Interaction attention and Entities attention can be well coordinated. In the fifth experiment (BERT + Int* + Ent* + MOL), we examine the influence of molecular structure on the model, we add molecular structure on the basis of the fourth experiment, the F1-score increases by 0.41% compared with the fourth experiment. Experimental results show that this method is effective, and also got the highest score (85.16%) currently compared to the existing model.

**Table 4:** Ablation experiment over our proposed model

Models	Precision (%)	Recall (%)	F1-score (%)
BERT	80.1	75.3	78.1
BERT + Int*	82.79	79.19	80.89
BERT + Ent*	85.04	82.73	83.76
BERT + Int* + Ent*	85.54	83.56	84.47
BERT + Int* + Ent* + MOL	**85**.**63**	**85**.**17**	**85**.**16**

Bold indicates the highest value of the measured metric in each comparison experiment

### Performance on fivefold cross validation

We used fivefold cross validation to further explore the stability of the experimental results and the practicability of our method. As can be seen from Table 5, in the experimental results, the Interaction attention vector we proposed played a strong role in promoting the baseline model. For each of the four relationship categories we focused on, Interaction attention vector contribute to the performance of the model. Molecular structure also performed well in most of the results, but the effect was not as strong as Interaction attention vector. Finally, the performance of the model is greatly improved after we add all the useful information, and the experimental results also show that our method has strong generalization ability and stability.

**Table 5:** F1-scores on fivefold cross-validated data set

	Methods	Adv. (%)	Effect (%)	Int (%)	Mech. (%)
Fold 1	Only-BERT	79.5	76.7	64.8	82.1
	+ Interaction_attention	83.9	77.0	70.3	84.6
	+ Int_attention + MOL	82.0	78.2	69.1	83.9
	+ All	**84**.**7**	**79**.**2**	**79**.**2**	**86**.**4**
Fold 2	Only-BERT	75.5	61.0	67.5	70.1
	+ Interaction_attention	80.3	**69**.**9**	70.2	77.6
	+ Int_attention + MOL	74.3	64.5	69.9	74.2
	+ All	**81**.**3**	69.7	**74**.**5**	**80**.**2**
Fold 3	Only-BERT	68.5	72.5	70.3	74.4
	+ Interaction_attention	**70**.**2**	79.9	69.3	82.1
	+ Int_attention + MOL	69.1	74.4	65.5	79.6
	+ All	70.1	**80**.**6**	**72**.**3**	**83**.**6**
Fold 4	Only-BERT	73.3	76.6	71.4	74.3
	+ Interaction_attention	**83**.**2**	75.5	**81**.**0**	75.0
	+ Int_attention + MOL	76.3	73.3	69.1	74.5
	+ All	82.4	**78**.**5**	79.5	**77**.**6**
Fold 5	Only-BERT	70.3	78.8	74.2	77.6
	+Interaction_attention	76.5	82.4	80.8	85.2
	+ Int_attention + MOL	77.5	80.4	75.2	79.2
	+ All	**79**.**3**	**85**.**8**	**81**.**3**	**86**.**6**

Bold indicates the highest value of the measured metric in each comparison experiment

## Discussion

### Error and analysis

In order to ensure the fairness of the results, we only adopt over-sampling and under-sampling processing for the training set. Although this is effective, the long-tail distribution of the test set itself cannot be solved, which is also a major feature and difficulty of biological data. In our test set, the data is very unevenly distributed. So, this was an important reason that affected the final results. Then the Interaction attention vector is mainly to better extract the information between two entities in a sentence, some sentences are very short, which can provide very little information to judge the relationship. Therefore, the performance can be improved by the Interaction attention vector is limited, and it is inevitable that there will be wrong classification results.

In addition, in the process of obtaining drug structures from drug names, we first need to derive the molecular formulas of drugs from medical knowledge base. In this process, some drugs could not find the corresponding molecular formulas. On the other hand, in the process of obtaining molecular structures from SMILES, we got a plane structure or wrong information, and the actual molecular structures of drugs is three-dimensional, which will cause us to lose a lot of information outside a plane structure. All these errors will have a great impact when transferred to the model.

### Interpretability

The performance of IMSE benefits from several major factors. (1) Interaction information contains key information of drug–drug interaction, and the introduction of Interaction attention vector improves the accuracy of features. (2) The introduction of entity information can fully extract the local information of entities in the feature space, which effectively reduces the interference of other entities to the model. (3) The addition of molecular structure effectively improves the richness of the feature space and provides information other than text.

## Conclusion

In this paper, we propose a DDI extraction model based on BioBERT to improve the performance of DDI extraction, termed IMSE. In our model, we use Interaction attention vector which we proposed to enhance the interaction information in sentences to better deal with relationship overlap problem. The molecular structure information we add can take advantage of knowledge that cannot be learned in text and can better characterize the drug feature space. In addition, we added entity attention vectors to enhance the weights around the entities so that the model can better capture the information around the drug entities without being distracted by other drugs. Comparative experiments on benchmark datasets showed that IMSE had a better predictive performance than existing prediction models, improving DDI identification. The performance of the model in the ablation experiment also shows that each part of the model is indispensable, which also provides new ideas for subsequent research, that is, we can obtain a better feature representation from the characteristics of the data.

Although our proposed approach exhibits promising performance for DDI extraction from biomedical literature, there is still some room to improve. In future work, we will continue to explore the characteristics of biomedical data and combine it with deep learning methods to better solve the problems of biomedical applications.

## Data Availability

The method details and implementation flow can be obtained from our Github repository: https://github.com/db-bionlp/IMSE.

## Abbreviations

CNN: Convolutional neural networks
RNN: Recurrent neural networks
LSTM: Long short-term memory networks
BERT: Bidirectional encoder representation from transformer
DDI: Drug drug interaction
DDIExtraction: Drug drug interaction extraction
BioBERT: Biomedical bidirectional encoder representation from transformer
SMILES: Simplified molecular input line entry specification
MGNN: Molecular graph neural networks
NLP: Natural language processing
GRU: Gate recurrent unit
